# Clinical Nonlinear Laser Imaging of Human Skin: A Review

**DOI:** 10.1155/2014/903589

**Published:** 2014-08-28

**Authors:** Riccardo Cicchi, Dimitrios Kapsokalyvas, Francesco Saverio Pavone

**Affiliations:** ^1^National Institute of Optics (INO), National Research Council (CNR), Largo E. Fermi 6, 50125 Florence, Italy; ^2^European Laboratory for Non-Linear Spectroscopy (LENS), University of Florence, Via Nello Carrara 1, 50019 Sesto Fiorentino, Italy; ^3^Department of Physics, University of Florence, Via Giovanni Sansone 1, 50019 Sesto Fiorentino, Italy

## Abstract

Nonlinear optical microscopy has the potential of being used *in vivo* as a noninvasive imaging modality for both epidermal and dermal imaging. This paper reviews the capabilities of nonlinear microscopy as a noninvasive high-resolution tool for clinical skin inspection. In particular, we show that two-photon fluorescence microscopy can be used as a diagnostic tool for characterizing epidermal layers by means of a morphological examination. Additional functional information on the metabolic state of cells can be provided by measuring the fluorescence decay of NADH. This approach allows differentiating epidermal layers having different structural and cytological features and has the potential of diagnosing pathologies in a very early stage. Regarding therapy follow-up, we demonstrate that nonlinear microscopy could be successfully used for monitoring the effect of a treatment. In particular, combined two-photon fluorescence and second-harmonic generation microscopy were used *in vivo* for monitoring collagen remodeling after microablative fractional laser resurfacing and for quantitatively monitoring psoriasis on the basis of the morphology of epidermal cells and dermal papillae. We believe that the described microscopic modalities could find in the near future a stable place in a clinical dermatological setting for quantitative diagnostic purposes and as a monitoring method for various treatments.

## 1. Introduction

The “gold standard” for tissue diagnostics is the histological examination, which is performed by means of white light optical microscopy on cryosectioned, processed, and labelled slices of tissue. Modern optics provides imaging tools for a noninvasive label-free deep imaging of skin that offer the potential for both tissue diagnostics and therapy follow-up* in vivo* and* in situ*, without the requirement of a biopsy.

Among these optical techniques, two-photon fluorescence (TPF) microscopy [1] is a high-resolution laser scanning imaging technique enabling deep optical imaging of tissues. TPF intrinsically offers several advantages with respect to other laser scanning imaging techniques, including higher spatial resolution, intrinsic optical sectioning capability, reduced photodamage and phototoxicity, and deeper penetration depth within biological tissues [2]. Further, since both cells and extracellular matrix intrinsically contain a variety of fluorescent molecules (NADH, tryptophan, keratins, melanin, elastin, cholecalciferol, and others), biological tissues can be imaged by TPF microscopy without any exogenously added probe [3–5]. By taking advantage of mitochondrial NADH fluorescence, TPF microscopy can be used for a morphological characterization of epithelia, as demonstrated by studies performed on* ex vivo* tissue samples [6, 7], fresh biopsies [8–12] and also* in vivo* on both animals [13] and humans [14–18]. Additional morphological information can be provided by second-harmonic generation (SHG) microscopy [19–29], which can be combined with TPF microscopy using the same laser source. In particular, while TPF reveals the distribution of endogenous fluorophores such as NADH, flavins, elastin, and others, SHG microscopy is offering the direct high-resolution imaging of collagen structures. SHG was already largely used for imaging anisotropic molecules inside cells [19, 20] and tissues [21, 23]. Collagen fibres produce a high SHG signal [22] with which they can be imaged inside skin dermis. Recently, SHG was also used for investigating collagen fibres orientation and their structural changes in healthy tissues as human dermis [10, 24, 28, 30, 31] or cornea [25, 27, 32] and in the tumour microenvironment [33–35]. Combined TPF-SHG microscopy represent a powerful tool for imaging skin dermis, since the main dermal components, collagen and elastin, can be imaged by SHG and TPF microscopy, respectively [4]. In particular, it has been used for monitoring collagen alteration in dermal disorders [28] or at the tumour-stroma interface [33–35], as well as for monitoring skin aging by measuring the collagen/elastic fibres content [36–38]. Fluorescence lifetime imaging microscopy (FLIM), when performed with nonlinear excitation, is an additional noninvasive microscopy technique enabling the identification of endogenous fluorescence species and their surrounding medium by measuring the decay rate of fluorescence emission [39, 40]. FLIM is useful to study protein localization [41] and fluorescent molecular environment [42]. FLIM was demonstrated to be a powerful technique able to provide functional information about tissue conditions [16, 17, 39, 40, 43–45]. It was successfully used to characterize tissues and to detect cellular differentiation inside epithelia as demonstrated by studies performed on cell cultures [46], fresh biopsies [8, 11, 12], and recently also* in vivo* [18]. Further, functional information on tissue conditions can be revealed by means of time-resolved analysis of NADH emission [46–48]. TPEF-FLIM has been previously applied to the study of the fluorescent properties of both normal and diseased skin [16, 17, 45] and has been demonstrated as an important tool to characterize skin layers specificity [8, 16].

In this paper, after having described materials and methods, we first show how it is possible to differentiate various epidermal layers* in vivo* by using TPF microscopy. In particular, the detection of skin autofluorescence allows direct imaging of cells and their morphological classification based on the cellular and nuclear sizes. Additional functional information, related to the metabolic conditions of cells, can be extracted by analysing the temporal decay of NADH fluorescence by means of FLIM. We found that cells located in the basal layer have the strongest metabolic activity, whereas the activity is reduced when moving towards the epidermal surface. Such approach can be used for characterizing epithelial tissues in various physiologic conditions and has the potential to detect pathologies in a very early stage, as demonstrated by studies performed on cell cultures [46, 49], fresh biopsies [8, 11, 12], and also* in vivo* [18]. In the second part of the paper, we show two different examples demonstrating that nonlinear microscopy can be successfully used for monitoring the effect of a laser-based treatment and for diagnosing and monitoring psoriasis. In particular, combined TPF and SHG microscopy were used* in vivo *for monitoring collagen remodeling after microablative fractional laser resurfacing and for characterizing psoriasis on the basis of the morphology of epidermal keratinocytes and dermal papillae. In the first example,* in vivo* nonlinear imaging was performed at the dermal level on the forearm of healthy subjects before and forty days after microablative fractional laser resurfacing treatment with the aim of characterizing collagen organization. Both qualitative and quantitative analyses demonstrated a stronger collagen synthesis and remodelling on older subjects, whereas the modifications were minimal on younger subjects. The second example focuses on the morphological characterization of both skin epithelium and papillary dermis in psoriasis. The morphological differences that can be observed between healthy and psoriatic skin are already well established by histopathological examination. In the example shown, these morphological differences were visualized* in vivo* by means of nonlinear imaging. In particular, in psoriasis we observed a drastically different morphology of epithelium with respect to healthy skin that includes a thickening of corneum layer, a disorganization of corneocytes, and a more sparse arrangement of keratinocytes. Differences were observed also at the dermal level in terms of an increased density and penetration depth of dermal papillae in psoriasis with respect to healthy skin. Morphological and architectural information were quantified and could be used for monitoring their course during a systemic treatment. The imaging modalities presented here represent promising tools to be used for both diagnostic and therapy follow-up purposes in dermatology and they could find a stable place in a clinical dermatological setting in the near future.

## 2. Materials and Methods

### 2.1. Experimental Setup

**Figure 1:**
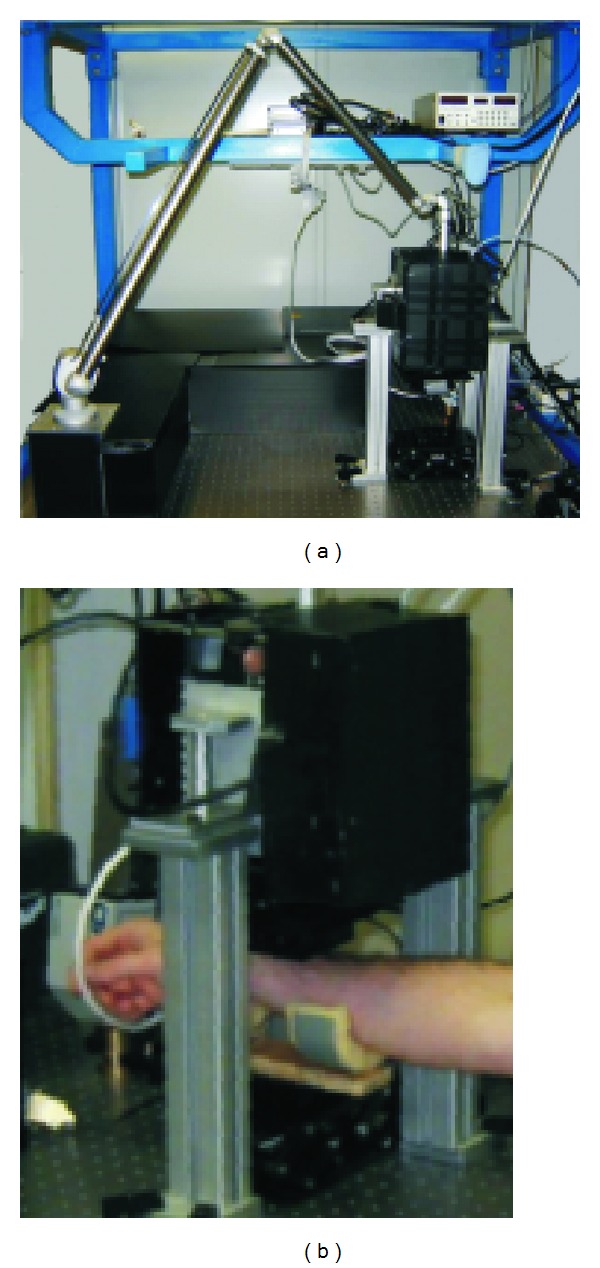
Experimental setup. (a) Photo of the custom nonlinear microscope experimental setup. (b) Detail of the forearm mounting stage.

The experimental setup for imaging is a custom-made compact flexible nonlinear laser scanning microscope to be used for* in vivo* skin inspection. The system is mainly composed of four parts: the laser source and the optical system on the bench; the 7-mirror articulated arm; the microscope head; and the multispectral detector (see Figure 1(a)). The laser source is a Chameleon Ultra II (Coherent, Santa Clara, CA, US) pulsed Ti:Sapphire laser emitting 140 fs pulses at 80 MHz repetition rate, tunable in the range 690 nm–1080 nm. The output beam passes through a polarization-based system for power adjustment, made with a rotating half-waveplate and a polarizer, before being collimated by a telescope. An electronic shutter SH05 (Thorlabs, Newton, NJ, US) allows minimizing the exposure of the sample. Laser beam is then coupled to the articulated arm (El.En. Group, Calenzano, Italy), which is made by three titanium tubes connected through couples of mirrors, mounted on ball-bearings-based 45-degree mounts. This solution allows moving the microscope head maintaining the laser beam aligned and centered inside the arm itself. The laser beam is sent to the microscope head, composed of three metallic plates that divide it into three levels. In the lower level we placed detectors electronics, galvo-mirrors driver C280 (Galvoline, Rome, Italy), and a stepping motor for focusing M-111.DG (Physik Insytrumente, Karlsruhe, Germany). In the midlevel (scanning plate) we have the scanning head G1222 (Galvoline, Rome, Italy), a beam expander, a dichroic mirror 685DCXRU (Chroma Technology Corporation, Rockingham, VT, US), and the objective lens. Fluorescence (and/or SHG) light is reflected by the dichroic mirror to the upper level (detection plate) through a hole in which a laser blocking filter E700SP-2P (Chroma Technology Corporation, Rockingham, VT, US) is inserted. On the upper level (detection plate), there are two detection systems. The former is composed of a photomultiplier tube H7422 (Hamamatsu, Hamamatsu City, Japan) and is based on photocurrent integration. The latter is based on single photon counting and is made by an objective lens Plan10x (Nikon, Tokyo, Japan) to collect light and to couple it into a multimode optical fibre (OF), connected to the multispectral detector (Multi-PMT). The insertion of a narrow band-pass filter HQ420BP (Chroma Technology Corporation, Rockingham, VT, US) in the detection path allows exclusively detecting SHG. The multispectral detector PML-Spec (Becker-Hickl GmbH, Berlin, Germany) is composed of a diffraction grating with 600 lines/mm and a 16-channel multianode photomultiplier strip with 200 ps FWHM pulses that allows time- and spectral-resolved detection. A special custom mount, placed on the optical bench, allows placing the forearm under the microscope for* in vivo* examination (see Figure 1(b)).

The experimental setup used for microablative fractional laser resurfacing is a SmartXide DOT (Deka, El.En. Group, Calenzano, Italy) providing pulsed illumination in a raster fashion in order to perform fractional ablation of skin. The experiments were performed using a laser power of 20 W, a pixel dwell time of 1 ms, and a spacing of 0.5 mm over a 15 mm by 15 mm treated area. The volunteers were treated in the volar part of the forearm.

### 2.2. Data Acquisition and Processing

Two objective lenses can be mounted on the microscope: PlanFluor 40x (Zeiss, Jena, Germany), oil immersion, NA1.3, and WD 0.16 mm, or XLUM 20x (Olympus Corporation, Tokyo, Japan), water immersion, NA0.9, and WD 2 mm. Acquisition and control are performed using a PC and two synchronized I/O boards: a PCI-MIO-16E (National Instruments, Austin, TX, US); a SPC-730 (Becker-Hickl GmbH, Berlin, Germany). The two boards are synchronized by an electronic timing board E-6502 (National Instruments, Austin, TX, US) providing a common trigger. The output settings are controlled by custom-made software developed in LabView 7.1 (National Instruments, Austin, TX, US) ambient. The visualization of the acquired FLIM images is accomplished using dedicated software SPCM 1.1 (Becker-Hickl GmbH, Berlin, Germany). Image pixels exponential fits, deconvolution, and fluorescence decay analyses are performed using the software SPC-Image 2.8 (Becker-Hickl GmbH, Berlin, Germany) using a double-exponential decay model. Graphs and histograms were prepared in Microcal Origin Pro 8.0 (OriginLab Co., Northampton, MA, US).

TPF images in the epidermis were typically acquired using the 40x objective lens, 740 nm as excitation wavelength, a resolution of 512 × 512 pixels (corresponding to a field of view of about 200 *μ*m), using a pixel dwell time of 20 *μ*s, and a power in the 20 mW–50 mW range, depending on the depth of recording. FLIM images were acquired using the same laser power and wavelength, with 128 × 128-pixel spatial resolution (corresponding to 100 *μ*m field of view), a pixel dwell time of 0.2 ms, and an integration time of approximately 40 s per image.

SHG images in the dermis were typically acquired using the 20x objective lens, 900 nm as excitation wavelength, a resolution of 512 × 512 pixels (corresponding to a field of view of about 400 *μ*m), using a pixel dwell time of 20 *μ*s, and a power in the 30 mW–60 mW range, depending on the depth of recording. FLIM images were acquired using the same laser power and wavelength, with 128 × 128-pixel spatial resolution (corresponding to 200 *μ*m field of view), a pixel dwell time of 0.2 ms, and an integration time of approximately 40 s per image. Spectral images were acquired in blocks of 16 spectral images (420 nm–620 nm spectral range) with 64 × 64-pixel spatial resolution, 64 *μ*m field of view dimension, using a pixel dwell time of 0.2 ms and an integration time of approximately 40 s per image block.

### 2.3. Examined Volunteers

The study on FLIM imaging of the epidermis (Section 3.1) included 2 healthy Caucasian male volunteers (31 and 32 years old). The study on fractional laser resurfacing (Section 3.2) included 9 healthy Caucasian volunteers (5 males, 4 females, age range: 27–79 years). Examined volunteers were divided into three groups according to their age: Group I (3 volunteers, age < 35 years), Group II (3 volunteers, 35 years < age < 60 years), and Group III (3 volunteers, age > 60 years). The study on psoriasis (Section 3.3) included 8 Caucasian volunteers, 5 healthy and 3 affected with psoriasis. All the studies were approved by the Institutional Review Board of the University of Florence and conducted according to the tenets of the Declaration of Helsinki. Written informed consents were obtained from all participants after detailed explanation of the study.

## 3. Results

### 3.1. *In Vivo* Imaging and Characterization of Epidermal Layers

**Figure 2:**
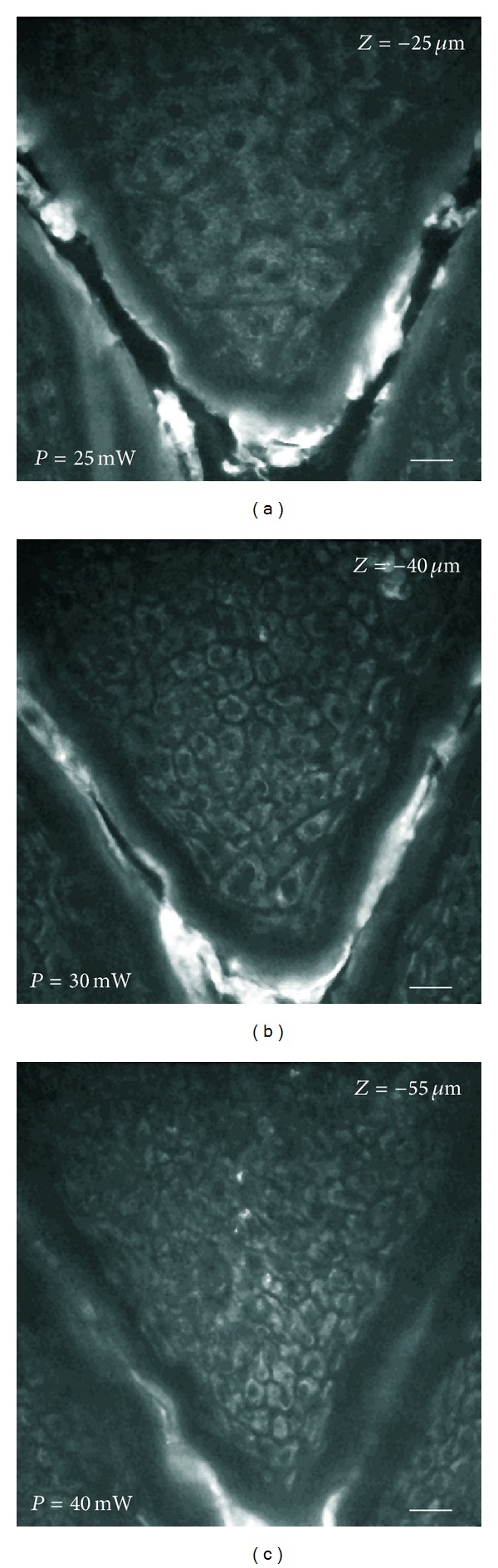
Morphological examination of epidermis. TPF image of NADH autofluorescence acquired from the epidermis of a healthy male volunteer at 25 *μ*m depth from skin surface (a), 40 *μ*m depth from skin surface (b), and 55 *μ*m depth from skin surface (c). The images are approximately corresponding to granular layer (a), spinous layer (b), and basal layer (c). Laser power, measured after the objective, is indicated in the down-left corner of each image. Excitation wavelength: 740 nm. Field of view: 200 *μ*m. Scale bar: 20 *μ*m.

*In vivo* imaging of the epidermis with subcellular spatial resolution can be performed by using TPF microscopy. Images of the various epidermal layers (Figures 2(a)–2(c)) were acquired by using a water immersion objective lens (NA 1.2) and an excitation wavelength of 740 nm. Signal was collected in the whole spectral range without any filtering in order to have a signal-to-noise ratio as high as possible. Cells are clearly distinguishable from the images and they appear with a fluorescent cytoplasm and a dark nucleus. Further, an analysis based on morphology enables discriminating various epidermal layers. In particular, our attention was focused on epidermal layers containing living cells. As expected, granular layer (Figure 2(a)) showed cells larger in dimension, with a characteristic content of highly fluorescent granuli, and emitting a lower average fluorescence with respect to other epidermal cells. Then, going deeper into skin (spinous layer), cells appear smaller in size, more round in shape and they are in average more fluorescent as well as tightly packed (Figure 2(b)) with respect to the upper layer. This trend is maintained when imaging deeper in the basal layer (Figure 2(c)) which contains the smaller epidermal cells having the largest metabolic activity. This feature corresponds to a higher fluorescent signal coming from this layer, as shown in Figure 2(c). Further, at this depth, images start exhibiting some extremely bright spots, probably corresponding to melanin granuli. Even if the probability of generating reactive oxygen species by prolonged multiphoton excitation inside skin has already been demonstrated to be not higher than normal sun exposure [50], at this depth we preferred to limit the acquisition only to few images because basal layer is the most absorbing region inside skin. Moreover, in order to limit the absorption to a minimum dose, images of epidermis were taken from the inner forearm which is one of the less pigmented regions of the body.

**Figure 3:**
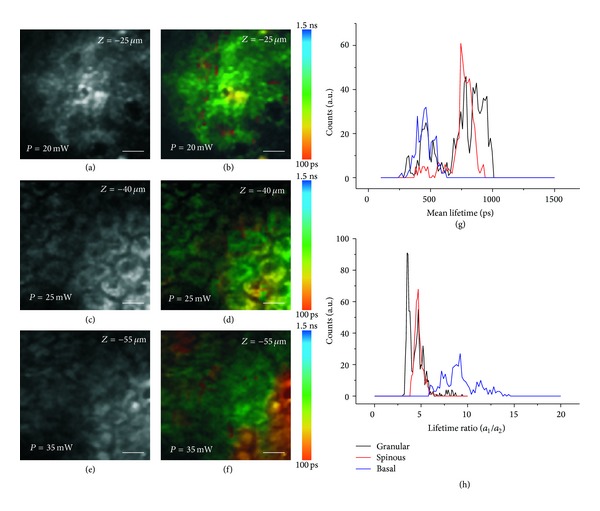
Layer-by-layer characterization of epidermis. TPF image of NADH autofluorescence and the corresponding FLIM images representing mean lifetime map acquired from the epidermis of a healthy male volunteer at 25 *μ*m depth from skin surface ((a), (b)), 40 *μ*m depth from skin surface ((c), (d)), and 55 *μ*m depth from skin surface ((e), (f)). The images are approximately corresponding to granular layer ((a), (b)), spinous layer ((c), (d)), and basal layer ((e), (f)). Images were acquired in the epidermis of a healthy male volunteer by using an excitation wavelength of 740 nm. Laser power, measured after the objective, is indicated in the down-left corner of each image. Field of view: 60 *μ*m. Scale bar: 6 *μ*m. Mean cellular lifetime distribution (g) and mean cellular lifetime components ratio (h) of the 3 different epidermal layers obtained after system response deconvolution and double-exponential fitting.

A layer-by-layer analysis of epidermis using FLIM was performed in skin of a healthy male volunteer who agreed to participate in the study. Mean lifetime distributions were calculated from images acquired at 20 *μ*m, 40 *μ*m, and 60 *μ*m depth from skin surface, approximately corresponding to granular, spinous, and basal layer, respectively (Figures 3(a)–3(f)). For these measurements, the excitation wavelength was set to 740 nm, which is good for exciting NADH autofluorescence, and the detection was in the 420 nm–620 nm range. Other tissue intrinsic fluorophores can be excited and detected in the same range because two-photon absorption spectra of intrinsic fluorophores are overlapping. The cellular mean lifetime distribution of the three layers is plotted in Figure 3 (g). The distributions of spinous and basal layers are well separated whereas the distribution obtained from the granular layer can be considered a mix of the previous two. The mean fluorescence lifetime for all the investigated layers is in the range of 1 ns, which is in good agreement with previous measurements performed on keratinocytes [11, 32, 33]. Even if other similar FLIM measurements on skin have already been performed in previous studies, they were more devoted to the differentiation between cells in healthy and cancer tissue such as healthy skin versus BCC [32] or melanocytic nevus versus melanoma [33]. Here we mainly focus on a layer-by-layer differentiation in healthy skin. The measured differentiation among skin layers in terms of fluorescence lifetime can be related to the differentiation in terms of proteins and cytokeratins content. In fact, in all the examined layers, even if the main contribution of tissue autofluorescence should arise from NADH, FAD, keratin, melanin, and cholecalciferol, the observed differences could be due not only to a different relative abundance of the molecules listed above, but also to the cytokeratin content in each layer. In particular, granular layer is characterized by the presence of loricrin and profilaggrin; spinous layer by cytokeratins 1 and 5; basal layer by cytokeratins 5 and 14. A spectroscopic and lifetime analysis on all these purified molecules would not help in clarifying this point because fluorescence lifetime of the same molecules should differ when measured in the tissue environment. For lifetime components ratio distributions, a different distribution was found for the three layers. In particular, as shown in Figure 3(h) the distribution center moves to higher value when increasing depth. If we consider that NADH autofluorescence is responsible for the main contribution to endogenous TPF signal, the ratio can be related with tissue metabolism in terms of bound-to-free NADH. In fact, fluorescence lifetime is able to determine if a fluorescent molecule is in its free or protein-bound state. NADH has a short lifetime in its free state and a much longer lifetime in its protein-bound state [47, 48]. The fluorescence lifetime of protein-bound NADH depends on the molecule to which it is bound and the changes in the binding site of NADH, connected with tumour development, can be potentially probed by measuring its lifetime. All these features can be used to optically monitor the metabolic state of a tissue and to potentially detect cancer in a very early stage on the basis of the fluorescence lifetime components ratio, as demonstrated by Skala and coauthors on cultivated living tissues [49, 51]. When moving from skin surface to the inside, epidermal cells differentiate: deeper located cells are smaller in dimension, younger in age and they have higher metabolic activity. The result shown in Figure 3(h) can be interpreted as a confirmation of this effect, because basal cells were found having the largest ratio, corresponding to a higher metabolic activity. Hence, a detailed characterization and differentiation of various epidermal layers, useful for diagnostics, can be obtained by analyzing the decay of NADH autofluorescence using FLIM. In particular, the mean fluorescence lifetime of NADH and the ratio of fast to slow fluorescence lifetime components can be taken as indicator of the metabolic state of cells. Considering that an altered metabolic activity of cells is very often precursor of a diseased state, these two parameters offer the potential to be used for diagnosing altered physiological conditions in a very early stage.

### 3.2. Noninvasive Follow-Up of Collagen Remodeling after Fractional Laser Resurfacing

**Figure 4:**
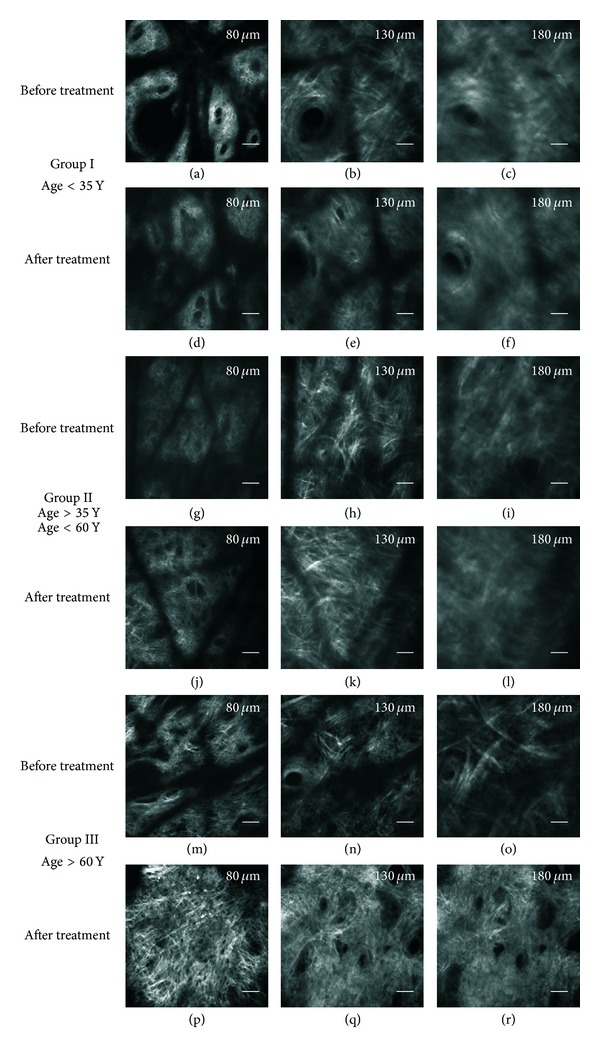
SHG imaging of collagen after laser resurfacing. SHG images of human dermis taken at 80 *μ*m, 130 *μ*m, and 180 *μ*m from skin surface on the inner forearm of healthy volunteers before and 40 days after microablative fractional laser resurfacing treatment. On the top, representative images of Group I (age < 35 years) taken before ((a), (b), and (c)) and 40 days after ((d), (e), and (f)) the treatment. In the middle, representative images of Group II (35 years < age < 60 years) taken before ((g), (h), and (i)) and 40 days after ((j), (k), and (l)) treatment. At the bottom, representative images of Group III (age > 60 years) taken before ((m), (n), and (o)) and 40 days after ((p), (q), and (r)) treatment. Field of view: 400 *μ*m. Scale bars: 40 *μ*m. Figure modified from [38].

A microscopic observation of the effects on collagen caused by microablative laser resurfacing treatment was performed by using SHG microscopy. SHG images were acquired in the dermis of volunteers at depths of 80 *μ*m, 130 *μ*m, and 180 *μ*m from skin surface. A qualitative nonblinded microscopic analysis was performed by visual examination of SHG images acquired immediately before and 40 days after treatment (Figure 4). The acquired SHG images were visually examined for extracting information on collagen fibres and amorphous component appearance that are related to skin aging. In particular, as the age increases we expect an increase in collagen fibres thickness and density, a decrease of the amorphous component (mainly composed of hyaluronic acid and glycosaminoglycans) that affects tissue hydration [52]. Collagen can be directly visualized on SHG images, whereas the increased scattering that gives to the images a more “cloudy” appearance can indirectly give an indication about the amorphous component abundance [38].

For Group I (age < 35 years), in the images acquired after treatment (Figures 4(d), 4(e), and 4(f)) we did not observe any significant modification with respect to the corresponding images acquired before treatment (Figures 4(a), 4(b), and 4(c)). Both the collagen and the amorphous component show a similar appearance before and after the treatment. For Group II (35 years < age < 60 years, Figures 4(g)–4(l)), we observed a slight increase of collagen fibres density after treatment; the amorphous component increases as demonstrated by a more “cloudy” appearance of the images. For Group III (age > 60 years, Figures 4(m)–4(r)), collagen fibres strongly increase in density, the amorphous component undergoes a drastic improvement, and both the epidermal thickness and the number of dermal papillae increase. In this group, all the observed features are in agreement with a rejuvenating effect. Even if the effectiveness of the treatment seems to increase with age, as confirmed by the following quantitative analysis, a better statistics on a larger number of volunteers would be beneficial to confirm this tendency.

**Figure 5:**
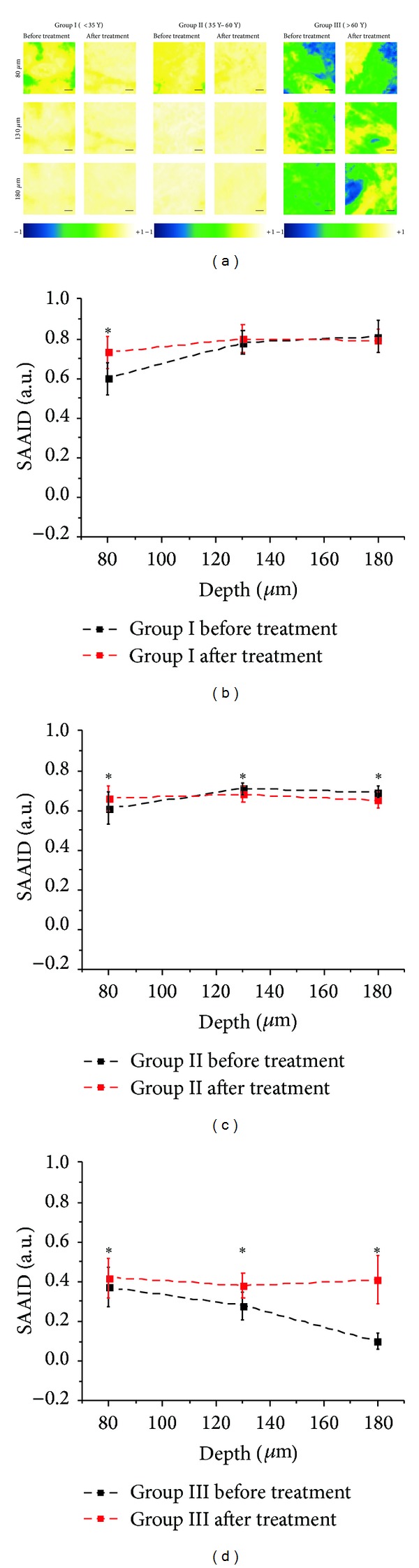
SAAID analysis of dermis after laser resurfacing. (a) SAAID images of human dermis at 80 *μ*m, 130 *μ*m, and 180 *μ*m from skin surface before and after microablative fractional laser resurfacing treatment. On the left, representative SAAID images of Group I (age < 35 years) taken before (left column) and 40 days after (right column) treatment. In the middle, representative SAAID images of Group II (35 years < age < 60 years) taken before (left column) and 40 days after (right column) treatment. On the right, representative SAAID images of Group III (age > 60 years) taken before (left column) and 40 days after (right column) treatment. Scale bars: 40 *μ*m. Color-coded scales for SAAID are plotted beneath each of the three groups of figures. SAAID score versus depth of recording for (b) Group I before (black) and after (red) treatment; (c) Group II before (black) and after (red) treatment; (d) Group III before (black) and after (red) treatment. Data are averaged on all the acquired data sets. The error bars correspond to the standard deviation of measured data. The SAAID mean values before and after treatment were found to be statistically different (indicated with ∗) or not statistically different (no ∗) at the 0.05 level after a two-sample statistical *t*-test (*P* = 0.05). Figure derived from [38].

A quantitative microscopic analysis, aimed at evaluating treatment effectiveness, was performed using the so-called second-harmonic to autofluorescence aging index of dermis (SAAID) [10, 36–38]. The SAAID defines a normalized ratio between SHG and TPF, so that it can be used for monitoring skin photoaging based on the ratio between collagen and elastic fibres. In fact, whereas collagen fibres should decrease with aging, elastic fibres should increase, causing a decrease of the SAAID with age. Hence, we expected an increase of the SAAID after treatment. SAAID images of the three examined groups are represented in Figure 5(a). SAAID analysis was performed on all the acquired spectral images, and the results were averaged and plotted in graphs (Figures 5(b), 5(c), and 5(d)). It has to be noted that before treatment the average SAAID level of Group III is always lower than that of the second group, which is in turn lower than that one of the first group, confirming that this parameter decreases with increasing age, as observed in other studies aimed at monitoring skin photoaging [36–38, 53, 54]. When comparing SAAID levels before and after treatment, the results show a negligible increase on young volunteers of Group I (age < 35 years, Figure 5(b)), a very small increase on Group II (35 years < age < 60 years, Figure 5(c)), and a strong increase on Group III (age > 60 years, Figure 5(d)). Even if the increase is small, in Group II the SAAID exhibits a statistically significant variation (after a two-sample statistical *t*-test at the 0.05 level) when comparing images before and after treatment. In Group III SAAID is increasing after treatment, exhibiting statistically significant variations (after a two-sample statistical *t*-test at the 0.05 level). This confirms the fact that the treatment has stronger effect on more aged subjects. This result is in agreement with the visual observation described in the previous section, where the difference between the youngest and the oldest volunteers in terms of treatment effectiveness was clearly distinguishable from the images. In conclusion, SAAID analysis allowed better delineation of treatment effectiveness versus age.

### 3.3. *In Vivo* Clinical Investigation of Psoriasis

The microscopic imaging of psoriatic skin was performed on the dorsal forearm of the volunteers [55]. Imaging of the epidermis and of the dermis was performed under different conditions, as reported in the Materials and Methods section. TPF images of the epidermis were acquired using the excitation wavelength of 740 nm, which is adequate for exciting NADH fluorescence, and a power in the 10 mW–40 mW range, depending on the depth of recording. SHG images of the dermis were acquired using the excitation wavelength of 900 nm and a power in the 20 mW–60 mW range, depending on the depth of recording.

**Figure 6:**
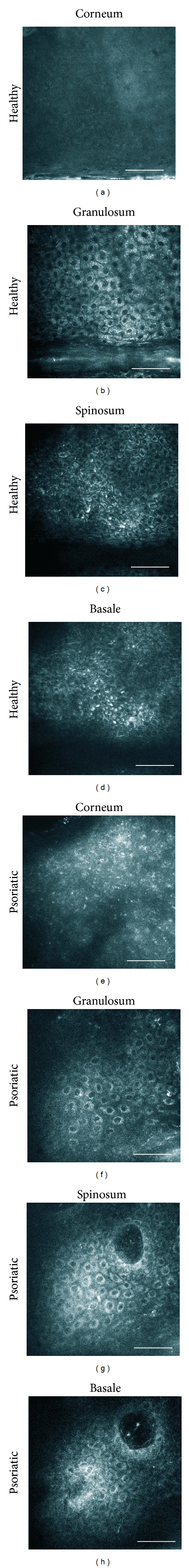
TPF imaging of healthy and psoriatic epidermis. Two-photon autofluorescence images of the layers of the epidermis. The images come from the dorsal forearm of a healthy ((a)–(d)) and a psoriatic ((e)–(h)) skin. Scale bars: 50 *μ*m. Figure modified from [55].

Two typical image stacks, respectively, from a healthy and psoriatic epidermis are shown in Figure 6. The images correspond to the acquisition from a 30-year-old healthy male (Figures 6(a)–6(d)) and from a 35-year-old female with psoriasis (Figures 6(e)–6(h)). Imaging starts at a depth located immediately below skin surface for safety and practical reasons. In healthy skin, the fluorescence originating from the corneum layer is very strong (Figure 6(a)) and it appears to be uniform without a characteristic morphology. On the other hand, in psoriatic skin the fluorescence level is lower and a characteristic punctuated pattern appears (Figure 6(e)). The physiological origin of this signal is still under evaluation. This atypical morphology of corneum layer is probably because psoriatic keratinocytes are not completely differentiated. This morphology was confirmed by the acquisitions in the other cases of psoriasis but it was never observed in healthy skin. Hence, it could be considered as a characteristic feature of psoriasis. At the depth of stratum granulosum, typical big cells with the characteristic granular morphology in the cytoplasm are found in healthy epidermis (Figure 6(b)), whereas this layer is very thin and in some cases is even absent in psoriatic skin. Here, psoriatic cells (Figure 6(f)) have a very small cytoplasmic area. Moving down to the stratum spinosum, in healthy skin cells are densely packed and have the characteristic spiny morphology (Figure 6(c)). This is a relatively extended layer with cellular morphology and size similar to those of the stratum granulosum, with the only difference of a more uniform fluorescence. On the other hand, at this depth psoriatic cells (Figure 6(g)) appear with a very small cytoplasmic area and bigger nuclei compared to healthy cells. The packing is very sparse with big distances between adjacent cells. In the healthy stratum basale (Figure 6(d)) cells have even smaller size, they are densely packed, and they emit a strong fluorescence. In psoriatic skin (Figure 6(h)), this layer is not clearly distinguishable in a single optical section due to its typical wavy morphology and deep epidermal proliferation. Nevertheless, it is possible to identify basal cells by looking around the formation of the dermal papillae. Here cells show a very small cytoplasmic area and their packing is even denser compared to stratum spinosum. Another characteristic feature of psoriatic skin at this depth is the presence of dermal papillae that infiltrate deep inside the epidermis (Figures 6(g)-6(h)). On the other hand, in healthy skin a papilla infiltrating inside the epidermis can be observed only occasionally and it never protrudes so near to the surface.

**Figure 7:**
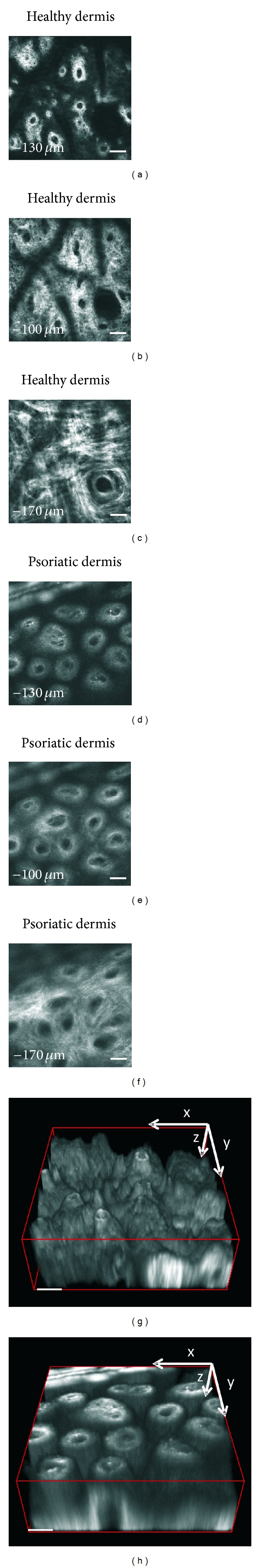
SHG imaging of healthy and psoriatic dermis. ((a)–(c)) SHG images from healthy papillary dermis. ((d)–(f)) SHG images from psoriatic papillary dermis. Scale bars: 50 *μ*m. ((g)-(h)) 3D reconstruction of the dermal layer of healthy (g) and psoriatic (h) skin lesion. The volumes are rotated 30° in the *y*-*z* plane. (g) The papillae in healthy skin are short, typically 30 *μ*m, and with small diameter, around 29 *μ*m. (h) Psoriatic papillae dimensions are bigger with typical lengths more than 100 *μ*m and a diameter of around 65 *μ*m. (Scale bars: 50 *μ*m.) Figure modified from [55].

Imaging of the papillary dermis was performed using SHG microscopy in both healthy and psoriatic skin. Three images from a typical image stack acquired from the dorsal forearm of a healthy 30-year-old male are presented in Figures 7(a)–7(c). Imaging starts at a depth of about 85 *μ*m from the surface and it goes deeper. In the first dermal layers, collagen fibres have a small diameter (around or less than 1 *μ*m), a curly appearance and they form a very complex and dense network. At this depth, dermal papillae dominate the images and their density is high. Dark regions around papillae are occupied by epidermal cells proliferating inside dermis. At a depth of 150 *μ*m from skin surface most of the dermal papillae disappear in the dense collagen network. When moving deeper, collagen fibres gradually increase in size and the collagen network appears with a better contrast. The network is less complex and the direction of the fibres is more ordered. At the depth of 180 *μ*m the quality of the image starts to degrade due to scattering. Three images from a typical image stack acquired from the dorsal forearm of a psoriatic 35-year-old female are presented in Figures 7(d)–7(f). Similarly to healthy skin, imaging starts at a depth of about 85 *μ*m from the surface and it goes deeper. By observing the formation of papillae in the first dermal layers, we noted that the density of papillae is higher compared to healthy skin. The presence of papillae is still evident also when moving deeper into the tissue, where the space around them starts to be filled with collagen. At a depth of 170 *μ*m from the skin surface, dermis starts having a similar morphology with respect to healthy skin. However, the fine collagen network of interwoven curly fibres below the dermoepidermal junction that is seen in healthy skin is not visible in psoriasis.

From the acquired images, it looks like the formation of papillae in psoriasis starts at depths around 170 *μ*m below the skin surface, whereas in healthy skin the formation starts at a depth of around 115 *μ*m. The characteristic feature of psoriatic skin (Figures 7(d)–7(f)) is the elongated dermal papillae. This feature becomes better visible in the 3D reconstruction of this part of the skin. Volume stacks with 5 *μ*m step are recorded and used to produce the 3D reconstruction. The result is presented in Figures 7(g) and 7(h) where the 3D reconstruction of the papillae is shown for both healthy and psoriatic skin. Examination of the 3D reconstruction revealed that papillae in psoriasis have a length of around 100 *μ*m, which is much longer than the length of papillae in healthy skin, which is around 30 *μ*m. In addition, in psoriasis dermal papillae are larger in diameter compared to healthy skin. A quantitative evaluation of this feature was obtained by measuring the cross-sectional surface occupied by dermal papillae at around 10 *μ*m below their tip in both healthy and psoriatic skin. Considering the fact that the shape of the cross-section of a papilla is closer to an ellipse than to a circle, we characterized the papilla size based on their surface rather than on their diameter. Then, a typical diameter was extrapolated by considering the size of the diameter of a circle that would occupy the same surface of the measured ellipse. This calculation provided the following results: a papilla in healthy skin had a mean surface of 650 ± 50 *μ*m^2^, approximately corresponding to a diameter of 29 ± 9 *μ*m; a papilla in psoriasis had a mean surface of 3300 ± 400 *μ*m^2^, approximately corresponding to a diameter of 64 ± 22 *μ*m. In conclusion, the length of the dermal papillae is at least 3 times longer in psoriasis and almost doubled in diameter compared to healthy skin.

## 4. Discussion

In this review, we highlighted the capability of nonlinear microscopy for clinical dermatological imaging of both epidermis and dermis* in vivo*. In particular, both cellular epidermis and dermal collagen morphology can be noninvasively imaged and characterized by means of TPF and SHG microscopy, respectively. These two techniques are able to noninvasively provide high-resolution images of skin morphology that can be used for diagnostic purposes.

Although skin morphology can be noninvasively imaged with high resolution by other laser scanning imaging techniques, such as confocal reflectance microscopy [56–68], the nonlinear approach offers several advantages with respect to the confocal reflectance. First, the nonlinear dependence of the signal on the excitation light intensity allows selectively exciting only molecules located in an extremely confined volume around the focal point. The direct consequence is an intrinsically high spatial resolution in comparison with other conventional microscopy techniques that employ similar excitation wavelengths. Second, a confocal pinhole rejecting out-of-focus light is not required in nonlinear microscopy, allowing for a 3D scanning of the specimen using a reduced exposure with respect to confocal microscopy. Third, every biological tissue intrinsically contains a certain amount of fluorescent molecules and SHG emitters that can be excited using nonlinear microscopy. This allows not only imaging the morphology of a biological tissue without adding any probe, but also extracting functional information. Finally, the use of particular contrast methods based on nonlinear excitation, such as SHG and FLIM, allows a more specific identification of molecular species in skin, such as collagen in dermis and other nucleotides in epidermis. The selective imaging and spectroscopy of these molecules can provide information about the physiologic condition of the tissue and hence they can be used for both diagnostic and therapy follow-up purposes.

As demonstrated in this review, additional characterization and differentiation of various epidermal layers, useful for diagnostics, can be obtained by analyzing the decay of NADH autofluorescence using FLIM. In particular, the mean fluorescence lifetime of NADH and the ratio of fast to slow fluorescence lifetime components can be taken as indicator of the metabolic state of cells. Considering that an altered metabolic activity of cells is very often precursor of a diseased state, these two parameters offer the potential to be used for diagnosing altered physiological conditions in a very early stage.

The potential clinical dermatological applications of nonlinear microscopy are not limited to skin diagnostics, but they extend also to treatment follow-up. In this review, we demonstrated the capability of nonlinear microscopy for imaging dermis* in vivo* after microablative fractional laser resurfacing treatment. In particular, for the first time nonlinear microscopy was used* in vivo* with the final goal of monitoring a laser-based treatment [38]. The obtained results have shown that nonlinear microscopy is able non-invasively providing a quantitative measurement of the efficacy of the resurfacing treatment. The production of new collagen, as well as an increase in the amount of dermal amorphous component, was found within 40 days from the laser treatment. The effects caused by the treatment can be evaluated qualitatively by visual examination of SHG images of collagen, and quantitatively by measuring the relative amount of SHG and TPF signals, through the SAAID index. The SAAID analysis demonstrated a strong treatment effectiveness in older subjects, whereas the effect was found to be negligible in young and minimal in middle-age subjects.

These nonlinear imaging techniques were additionally used for* in vivo* revealing the characteristic micromorphology of psoriasis at both epidermal and dermal levels. In the epidermis, psoriatic cells have very small cytoplasmic area and they are sparsely packed compared to healthy cells. Further, the more pronounced epidermal proliferation and the dilated papillae of the dermis, typical of psoriasis, were imaged at high resolution. A 3D reconstruction of dermal papillae revealed the great difference of psoriatic skin morphology, where dilated and elongated dermal papillae can be observed. A quantitative measurement demonstrated that the length of dermal papillae is 60% longer in psoriasis and almost doubled in size compared to healthy skin. Imaging and characterization of psoriasis with such a detail is not required for diagnostics. However, during the past years there has been a great effort in the research for the treatment of this disease [69–71]. These therapies have risks that could be reduced if the effect of a treatment can be timely assessed. Currently the severity of psoriasis and the effect of a treatment are evaluated by the Psoriasis Area and Severity Index (PASI) [72]. A potential quantitative monitoring of a treatment effect on psoriasis could be based on the measurement of the nucleus to cytoplasm ratio in epidermis and on papillae size in dermis. Correlation of the measured values with the well-established PASI during the course of a treatment could provide a means of comparison and evaluation of the proposed methodologies. Apart from monitoring the effect of an experimental treatment, the presented imaging techniques could be used for the personalization of existing treatments.

## 5. Conclusion

In conclusion, the methodologies described here could become a powerful tool to be used in a dermatological clinic for both early diagnosis and therapy follow-up purposes. New emerging technologies for ultrafast pulsed laser sources, potentially cheaper than the usual solid state Ti:Sapphire oscillator, can help nonlinear laser scanning microscopy to become more and more popular among medical doctors with the final goal of being recognized as a standard clinical imaging method. On the basis of the results described here and of several other successful dermatological applications experienced* in vivo* by means of nonlinear microscopy in recent years [16–18, 37, 38, 45, 53, 54], we believe that in the near future nonlinear laser scanning microscopy will find a stable place in the clinical dermatological setting.

## Abbreviations

TPF: Two-photon fluorescence
SHG: Second-harmonic generation
FLIM: Fluorescence lifetime imaging microscopy
SAAID: Second-harmonic to autofluorescence aging index of dermis.

## References

[1] Denk W, Strickler JH, Webb WW (1990). Two-photon laser scanning fluorescence microscopy. *Science*.

[2] Helmchen F, Denk W (2005). Deep tissue two-photon microscopy. *Nature Methods*.

[3] Zoumi A, Yeh A, Tromberg BJ (2002). Imaging cells and extracellular matrix in vivo by using second-harmonic generation and two-photon excited fluorescence. *Proceedings of the National Academy of Sciences of the United States of America*.

[4] Zipfel WR, Williams RM, Christiet R, Nikitin AY, Hyman BT, Webb WW (2003). Live tissue intrinsic emission microscopy using multiphoton-excited native fluorescence and second harmonic generation. *Proceedings of the National Academy of Sciences of the United States of America*.

[5] Zipfel WR, Williams RM, Webb WW (2003). Nonlinear magic: multiphoton microscopy in the biosciences. *Nature Biotechnology*.

[6] Laiho LH, Pelet S, Hancewicz TM, Kaplan PD, So PTC (2005). Two-photon 3-D mapping of ex vivo human skin endogenous fluorescence species based on fluorescence emission spectra. *Journal of Biomedical Optics*.

[7] Cicchi R, Pavone FS (2011). Non-linear fluorescence lifetime imaging of biological tissues. *Analytical and Bioanalytical Chemistry*.

[8] Cicchi R, Massi D, Sestini S (2007). Multidimensional non-linear laser imaging of Basal Cell Carcinoma. *Optics Express*.

[9] Paoli J, Smedh M, Wennberg A, Ericson MB (2008). Multiphoton laser scanning microscopy on non-melanoma skin cancer: morphologic features for future non-invasive diagnostics. *Journal of Investigative Dermatology*.

[10] Cicchi R, Sestini S, de Giorgi V, Massi D, Lotti T, Pavone FS (2008). Nonlinear laser imaging of skin lesions. *Journal of Biophotonics*.

[11] Cicchi R, Crisci A, Cosei A (2010). Time- and spectral-resolved two-photon imaging of healthy bladder mucosa and carcinoma in situ. *Optics Express*.

[12] Cicchi R, Sturiale A, Nesi G (2013). Multiphoton morpho-functional imaging of healthy colon mucosa, adenomatous polyp and adenocarcinoma. *Biomedical Optics Express*.

[13] Palero JA, de Bruijn HS, van der Ploeg van den Heuvel A, Sterenborg HJCM, Gerritsen HC (2007). Spectrally resolved multiphoton imaging of in vivo and excised mouse skin tissues. *Biophysical Journal*.

[14] Masters BR, So PTC, Gratton E (1998). Optical biopsy of in vivo human skin: multi-photon excitation microscopy. *Lasers in Medical Science*.

[15] Masters BR, So PTC (2001). Confocal microscopy and multi-photon excitation microscopy of human skin in vivo. *Optics Express*.

[16] König K, Riemann I (2003). High-resolution multiphoton tomography of human skin with subcellular spatial resolution and picosecond time resolution. *Journal of Biomedical Optics*.

[17] Dimitrow E, Riemann I, Ehlers A (2009). Spectral fluorescence lifetime detection and selective melanin imaging by multiphoton laser tomography for melanoma diagnosis. *Experimental Dermatology*.

[18] König K (2008). Clinical multiphoton tomography.. *Journal of Biophotonics*.

[19] Moreaux L, Sandre O, Charpak S, Blanchard-Desce M, Mertz J (2001). Coherent scattering in multi-harmonic light microscopy. *Biophysical Journal*.

[20] Campagnola PJ, Loew LM (2003). Second-harmonic imaging microscopy for visualizing biomolecular arrays in cells, tissues and organisms. *Nature Biotechnology*.

[21] Campagnola PJ, Millard AC, Terasaki M, Hoppe PE, Malone CJ, Mohler WA (2002). Three-dimensional high-resolution second-harmonic generation imaging of endogenous structural proteins in biological tissues. *Biophysical Journal*.

[22] Roth S, Freund I (1979). Second harmonic generation in collagen. *The Journal of Chemical Physics*.

[23] Williams RM, Zipfel WR, Webb WW (2005). Interpreting second-harmonic generation images of collagen I fibrils. *Biophysical Journal*.

[24] Stoller P, Reiser KM, Celliers PM, Rubenchik AM (2002). Polarization-modulated second harmonic generation in collagen. *Biophysical Journal*.

[25] Matteini P, Ratto F, Rossi F (2009). Photothermally-induced disordered patterns of corneal collagen revealed by SHG imaging. *Optics Express*.

[26] Vanzi F, Sacconi L, Cicchi R, Pavone FS (2012). Protein conformation and molecular order probed by second-harmonic-generation microscopy. *Journal of Biomedical Optics*.

[27] Matteini P, Cicchi R, Ratto F (2012). Thermal transitions of fibrillar collagen unveiled by second-harmonic generation microscopy of corneal stroma. *Biophysical Journal*.

[28] Cicchi R, Kapsokalyvas D, de Giorgi V (2010). Scoring of collagen organization in healthy and diseased human dermis by multiphoton microscopy. *Journal of Biophotonics*.

[29] Cicchi R, Matthäus C, Meyer T (2014). Characterization of collagen and cholesterol deposition in atherosclerotic arterial tissue using non-linear microscopy. *Journal of Biophotonics*.

[30] Yasui T, Tohno Y, Araki T (2004). Characterization of collagen orientation in human dermis by two-dimensional second-harmonic-generation polarimetry. *Journal of Biomedical Optics*.

[31] Sun Y, Chen W, Lin S (2006). Investigating mechanisms of collagen thermal denaturation by high resolution second-harmonic generation imaging. *Biophysical Journal*.

[32] Han M, Giese G, Bille JF (2005). Second harmonic generation imaging of collagen fibrils in cornea and sciera. *Optics Express*.

[33] Brown E, McKee T, DiTomaso E (2003). Dynamic imaging of collagen and its modulation in tumors *in vivo* using second-harmonic generation. *Nature Medicine*.

[34] Lin SJ, Jee S, Kuo C (2006). Discrimination of basal cell carcinoma from normal dermal stroma by quantitative multiphoton imaging. *Optics Letters*.

[35] Provenzano PP, Eliceiri KW, Campbell JM, Inman DR, White JG, Keely PJ (2006). Collagen reorganization at the tumor-stromal interface facilitates local invasion. *BMC Medicine*.

[36] Lin SJ, Wu R, Tan H (2005). Evaluating cutaneous photoaging by use of multiphoton fluorescence and second-harmonic generation microscopy. *Optics Letters*.

[37] Koehler MJ, König K, Elsner P, Bückle R, Kaatz M (2006). In vivo assessment of human skin aging by multiphoton laser scanning tomography. *Optics Letters*.

[38] Cicchi R, Kapsokalyvas D, Troiano M (2013). In vivo non-invasive monitoring of collagen remodelling by two-photon microscopy after micro-ablative fractional laser resurfacing. *Journal of Biophotonics*.

[39] Tadrous PJ (2000). Methods for imaging the structure and function of living tissues and cells: 2. Fluorescence lifetime imaging. *Journal of Pathology*.

[40] Tadrous PJ, Siegel J, French PMW, Shousha S, Lalani E, Stamp GWH (2003). Flourescence lifetime imaging of unstained tissues: early results in human breast cancer. *The Journal of Pathology*.

[41] Chen Y, Periasamy A (2004). Characterization of two-photon excitation fluorescence lifetime imaging microscopy for protein localization. *Microscopy Research and Technique*.

[42] Hanson KM, Behne MJ, Barry NP, Mauro TM, Gratton E, Clegg RM (2002). Two-photon fluorescence lifetime imaging of the skin stratum corneum pH gradient. *Biophysical Journal*.

[43] Bastiaens PIH, Squire A (1999). Fluorescence lifetime imaging microscopy: spatial resolution of biochemical processes in the cell. *Trends in Cell Biology*.

[44] Cubeddu R, Pifferi A, Taroni P (1999). Fluorescence lifetime imaging: an application to the detection of skin tumors. *IEEE Journal on Selected Topics in Quantum Electronics*.

[45] Dimitrow E, Ziemer M, Koehler MJ (2009). Sensitivity and specificity of multiphoton laser tomography for in Vivo and ex vivo diagnosis of malignant melanoma. *Journal of Investigative Dermatology*.

[46] Skala MC, Riching KM, Bird DK (2007). In vivo multiphoton fluorescence lifetime imaging of protein-bound and free nicotinamide adenine dinucleotide in normal and precancerous epithelia. *Journal of Biomedical Optics*.

[47] Lakowicz JR, Szmacinski H, Nowaczyk K, Johnson ML (1992). Fluorescence lifetime imaging of free and protein-bound NADH. *Proceedings of the National Academy of Sciences of the United States of America*.

[48] Li D, Zheng W, Qu JY (2008). Time-resolved spectroscopic imaging reveals the fundamentals of cellular NADH fluorescence. *Optics Letters*.

[49] Skala MC, Riching KM, Gendron-Fitzpatrick A (2007). In vivo multiphoton microscopy of NADH and FAD redox states, fluorescence lifetimes, and cellular morphology in precancerous epithelia. *Proceedings of the National Academy of Sciences of the United States of America*.

[50] Fischer F, Volkmer B, Puschmann S (2008). Risk estimation of skin damage due to ultrashort pulsed, focused near-infrared laser irradiation at 800 nm. *Journal of Biomedical Optics*.

[51] Skala MC, Riching KM, Bird DK (2007). *In vivo* multiphoton fluorescence lifetime imaging of protein-bound and free nicotinamide adenine dinucleotide in normal and precancerous epithelia. *Journal of Biomedical Optics*.

[52] Ghersetich I, Lotti T, Campanile G, Grappone C, Dini G (1994). Hyaluronic acid in cutaneous intrinsic aging. *International Journal of Dermatology*.

[53] Koehler MJ, Hahn S, Preller A (2008). Morphological skin ageing criteria by multiphoton laser scanning tomography: non-invasive in vivo scoring of the dermal fibre network. *Experimental Dermatology*.

[54] Koehler MJ, Preller A, Kindler N (2009). Intrinsic, solar and sunbed-induced skin aging measured in vivo by multiphoton laser tomography and biophysical methods. *Skin Research and Technology*.

[55] Kapsokalyvas D, Cicchi R, Bruscino N (2014). In-vivo imaging of psoriatic lesions with polarization multispectral dermoscopy and multiphoton microscopy. *Biomedical Optics Express*.

[56] González S, Rajadhyaksha M, Rubinstein G, Anderson RR (1999). Characterization of psoriasis in vivo by reflectance confocal microscopy. *Journal of Medicine*.

[57] Huzaira M, Rius F, Rajadhyaksha M, Anderson RR, González S (2001). Topographic variations in normal skin, as viewed by *in vivo* reflectance confocal microscopy. *Journal of Investigative Dermatology*.

[58] González S, Tannous Z (2002). Real-time, in vivo confocal reflectance microscopy of basal cell carcinoma. *Journal of the American Academy of Dermatology*.

[59] Wang LT, Demirs JT, Pathak MA, González S (2002). Real-time, in vivo quantification of melanocytes by near-infrared reflectance confocal microscopy in the guinea pig animal model. *Journal of Investigative Dermatology*.

[60] González S, Gilaberte-Calzada Y, González-Rodríguez A, Torres A, Mihm MC (2004). In vivo reflectance-mode confocal scanning laser microscopy in dermatology. *Advances in Dermatology*.

[61] Yamashita T, Kuwahara T, González S, Takahashi M (2005). Non-invasive visualization of melanin and melanocytes by reflectance-mode confocal microscopy. *Journal of Investigative Dermatology*.

[62] Agero ALC, Busam KJ, Benvenuto-Andrade C (2006). Reflectance confocal microscopy of pigmented basal cell carcinoma. *Journal of the American Academy of Dermatology*.

[63] Scope A, Benvenuto-Andrade C, Agero AC (2007). In vivo reflectance confocal microscopy imaging of melanocytic skin lesions: consensus terminology glossary and illustrative images. *Journal of the American Academy of Dermatology*.

[64] González S, Gilaberte-Calzada Y (2008). In vivo reflectance-mode confocal microscopy in clinical dermatology and cosmetology. *International Journal of Cosmetic Science*.

[65] González S (2009). Confocal reflectance microscopy in dermatology: promise and reality of non-invasive diagnosis and monitoring. *Actas Dermo-Sifiliograficas*.

[66] Ulrich M, Lange-Asschenfeldt S, Gonzalez S (2012). Clinical applicability of in vivo reflectance confocal microscopy in dermatology. *Giornale Italiano di Dermatologia e Venereologia*.

[67] Ulrich M, Lange-Asschenfeldt S, Gonzalez S (2012). The use of reflectance confocal microscopy for monitoring response to therapy of skin malignancies. *Dermatology Practical & Conceptual*.

[68] Venturini M, Arisi M, Zanca A (2014). In vivo reflectance confocal microscopy features of cutaneous microcirculation and epidermal and dermal changes in diffuse systemic sclerosis and correlation with histological and videocapillaroscopic findings. *European Journal of Dermatology*.

[69] Lowes MA, Bowcock AM, Krueger JG (2007). Pathogenesis and therapy of psoriasis. *Nature*.

[70] Roberson EDO, Bowcock AM (2010). Psoriasis genetics: breaking the barrier. *Trends in Genetics*.

[71] Weger W (2010). Current status and new developments in the treatment of psoriasis and psoriatic arthritis with biological agents. *British Journal of Pharmacology*.

[72] Spuls PI, Lecluse LLA, Poulsen MNF, Bos JD, Stern RS, Nijsten T (2010). How good are clinical severity and outcome measures for psoriasis: quantitative evaluation in a systematic review. *Journal of Investigative Dermatology*.

